# Morphogenetic Variability as Potential Biomarker of Neurogenic Lesion Degree in Children with Spina Bifida

**DOI:** 10.3390/healthcare8010068

**Published:** 2020-03-24

**Authors:** Ivana Petronic, Dragoslav Marinkovic, Dejan Nikolic, Dragana Cirovic, Zoran Golubovic, Filip Milanovic, Suzana Cvjeticanin

**Affiliations:** 1 Physical Medicine and Rehabilitation Department, University Children’s Hospital, 11000 Belgrade, Serbia; ivana.pm@live.com (I.P.); denikol27@gmail.com (D.N.); cirovicdragana@yahoo.com (D.C.); 2 Faculty of Medicine, University of Belgrade, 11000 Belgrade, Serbia; dr.dejan.nikolic@outlook.com (Z.G.); filipmilanovic333@gmail.com (F.M.); 3 Serbian Academy of Sciences and Arts, Department of Chemical and Biological Sciences, 11000 Belgrade, Serbia; mmarink@eunet.rs; 4 Pediatric Surgery Department, University Children’s Hospital, 11000 Belgrade, Serbia; 5 Institute for Human Genetics, Faculty of Medicine, University of Belgrade, 11000 Belgrade, Serbia

**Keywords:** spina bifida, neurogenic lesion, genetic homozygosity, genetic variability

## Abstract

Aims. In this study we analyzed the degree of genetic homozygosity among spina bifida patients with different degrees of neurogenic lesion (*N* = 82), as well as their clinical and neurological characteristics, compared to healthy control individuals (*N* = 100). Methods. According to clinical and electromyographic findings, we separately assessed the type of neurogenic lesion (paresis or paralysis). Regarding the degree of neurogenic lesion, patients were classified into three groups: mild, moderate and severe. We analyzed six muscles. For assessing the degree of individual genetic homozygosity, we tested the presence and distribution of 15 homozygous recessive characteristics (HRC). Results. The predominant type of neurogenic lesion was paresis. Every third evaluated muscle was affected in the group with mild neurogenic lesion, while more than half were affected in the group with severe neurogenic lesion. The average values of HRCs among different groups of patients and the control showed the population-genetic differences that exist among them (control $\bar{x}$_HRC/15_ = 3.0 ± 0.2; mild $\bar{x}$_HRC/15_ = 3.6 ± 0.2; moderate $\bar{x}$_HRC/15_ = 4.8 ± 0.3; severe neurogenic lesion $\bar{x}$_HRC/15_ = 5.0 ± 0.3). Conclusions. Spina bifida patients have a significant increase of recessive homozygosity and a decreased variability compared to the control group. As neurogenic lesions are more severe, more affected muscles are present, as well as the increase of individual recessive homozygosity.

## 1. Introduction

Spinal dysraphism (spina bifida—SB) is congenital malformation of the spine, associated with incomplete neural tube closure during neurulation [1]. The etiology that governs formation of SB is still unknown, but there are studies implicating multifactorial origin [1,2]. Genetic components, both on populational and molecular levels, have been evaluated in the patients with SB [1,3,4,5]. Additionally, the environmental and maternal factors, along with folate and homocisteine metabolism, are studied as potential factors in the etiopathogenesis of SB [1,6,7,8]. 

Numerous research studies on genome-wide association study (GWAS), using human single-nucleotide polymorphism (SNP), have not given sufficiently conclusive results [9]. The selection of neural tube defects risk candidate genes was limited by the knowledge of neural tube formation during early embryogenesis processes [9]. However, so far, proposed genes that are involved in the determination of susceptibility to SB are 1p13, 6q27, 17q11.20q12 (OMIM number 182940), followed by 1p36.3, 1q43, 5p15.3-p15.2 and 14q24 (OMIM number 601634) [10]. 

The studied homozygous characters (HRCs) are obviously controlled by genes located on different human chromosomes, therefore they could be considered genetic markers on these chromosomes, as well as on numerous surrounding genes controlling different fitness elements. The recessive homozygosity amount that is established by performed HRC-test is practically an estimation of genetic loads present in any specific sample of humans. Our numerous studies considering the HRC-test showed the reliability of this methodology in determining eventual changes and differences in certain subpopulation groups [11,12,13,14,15,16].

Spina bifida can be presented as spina bifida occulta (SBO) and spina bifida aperta (SBA). Spinal dysraphism can cause malformations on bones and extremities, with reduced mobility and bowel/bladder function impairments [1,17].

It is often clinically difficult to classify the neurological impairments by neurological examination solely, therefore electrodiagnosis as a diagnostic tool may give insights for the more reliable postulation of patients’ clinical conditions [18,19]. 

Even though numerous research articles are published, describing the role of potential risk factors on the development of SB as an independent entity, there are insufficient data regarding the potential influence of genetic or other factors on the degree of neurogenic lesion in patients with SB. Therefore, the aim of our study was to analyze the degree of individual genetic recessive homozygosity and variability in the groups of SB patients with different degrees of neurogenic lesions, compared to a control group. Additionally, we wanted to estimate the frequencies of the clinical and neurological characteristics of these patients.

## 2. Methods

### 2.1. Study Group

We evaluated 122 children with spina bifida in the lumbo-sacral region that were examined and treated at the University Children’s Hospital in Belgrade in 2009–2018. From the tested sample, 82 of them fulfilled the inclusion criteria for the study (presence of neurogenic lesion). For confirmation and classification of SB, we used clinical examination and imaging techniques. Regarding clinical symptoms, patients were classified into two groups: a group with SBO and a group with SBA. 

### 2.2. Study Parameters

According to the clinical and electromyographic findings, we separately assessed type and degree of neurogenic lesion. All patients were categorized into two groups regarding type of neurogenic lesion: group with lower extremities’ paresis and group with lower extremities’ paralysis. Regarding the degree of neurogenic lesion, patients were classified into three groups (mild, moderate and severe).

In order to assess the degree of neurogenic lesion, we performed a neurophysiological examination that included electromyography (EMG) of certain muscles in at least four areas, to optimally increase the chance of abnormalities’ detection. For the evaluated muscles activity assessment by EMG needle (concentric needle electrode), we used a standardized procedure that included the analysis of insertional and spontaneous activity, as well as voluntary motor-unit action potentials (MUAP), with a voluntary contraction pattern [20]. The parameters from MUAP potentials that were analyzed are: amplitude (grading from 1+ to 3+), presence of polyphasia (grading from 1+ to 3+) and potentials duration (grading from 1+ to 3+). For clinical interpretation, we evaluated muscle strength using a muscle manual test (MMT), where grading was between 0 to 5 [21].

We analyzed six muscles that correspond to the anatomical region of lumbosacral spine level: Outer anal sphincter (OAS), rectus femoris (RF), tibialis anterior (TA), gastrocnemius medialis (GM), extensor digiti brevi (EDB) and pollicis longus (PL). Only patients with positive findings on EMG were included. 

According to the clinical presentation of MMT, we defined mild neurogenic lesion as paresis of certain muscle groups on lower extremities (MMT grade 4), with a mild degree of neurological dysphunction on neurophysiological examination (absent spontaneous activity with neuropathic MUAP up to 1+ for amplitude, polyphasia presence and duration, and decreased recruitment up to 1-). Moderate neurogenic lesion, due to clinical presentation, was defined as moderate complete paresis of muscles on lower extremities (MMT grades 2 and 3), with a moderate degree of neurological dysphunction on neurophysiological examination (absent spontaneous activity with neuropathic MUAP from 2+ to 3+ for amplitude, polyphasia presence and duration, and decreased recruitment from 2− to 3−). Severe neurogenic lesion was defined as the partial or complete paralysis of muscles on lower extremities (MMT grades 1 and 0), with a severe degree on neurological dysphunction on neurophysiological examination (absent spontaneous activity with 1–2 MUAPs on voluntary muscle contraction or no voluntary activity).

For the estimation of the degree of genetic homozygosity, we tested the presence of 15 homozygous recessive characteristics (HRC) using the HRC-test [11]. The HRC-test [11,12,13,14,15,16] has been applied to determine the proportion of such clearly expressed characteristics in every individual as markers of different chromosomes. 

The control sample consisted of 100 randomly chosen children from the local population, belonging to the same age group (3-16 years of age), same ethnicity and from the same locality.

We tested 10 homozygous recessive characteristics on the head region, which are; blue eyes (gene location at 19p13.1-q13.11, OMIM number 227240), attached ear lobe (OMIM number 128900), continuous hair line (OMIM number 194000), straight hair (1q21.3, OMIM number 139450), soft and blond hair (gene location 15q12, 15q13, OMIM number 227220; 14q32.1, OMIM number 210750; 12q21.3, OMIM number 611664; 11q13.3, OMIM number 612267), double hair whorl, opposite hair whorl orientation (OMIM number 139400), color blindness (gene location at Xq28, OMIM number 303800) and ear without Darwinian knot. Five of the tested recessive traits on extremities included: proximal thumb extensibility, index finger longer (males)/ shorter (females) than the ring finger (OMIM number 136100), left-handedness (gene location at 2p12–q22, OMIM number 139900), right thumb over left thumb (OMIM number 139800) and hand clasping pattern (OMIM number 139800) [10].

In this study, comparative analyses were made by the same person, with equal criteria for determining extremely pronounced homozygously recessive characteristics in tested groups of observed individuals, excluding subjective criteria. The person that tested the presence of HRCs was unaware of the type of SB, as well as the degree of neurogenic lesion in evaluated individuals. For the assessment of rater reliability, the trained person performed series of 3 testing, along with another qualified and experienced tester on the control sample of individuals. Both the trainee person and qualified tester were unaware of another person’s findings. The interrater reliability testing was graded as: values ≤ 0 - no agreement, 0.01–0.20—none to slight, 0.21–0.40—fair, 0.41–0.60—moderate, 0.61–0.80—substantial, and 0.81–1.00—almost perfect agreement [22].

The obtained results in more than two decades of using HRC-test show a similar range of variation, so the performance of the test by one trained person, in this particular study, showed exceptional reliability [16].

### 2.3. Statistical Analysis

To present the frequency of certain groups of evaluated clinical and neurological characteristics of SB patients, as well as of different forms of neurogenic lesion, we used whole numbers (N) and percentages. Mean values (MV) and standard error of mean (SEM) were used to present descriptions of HRCs, while mean values (MV) and standard deviation (SD) were used to present descriptions of evaluated muscles. Interrater reliability was presented as Cohens kappa value (Table 1) [22]. A Chi squared test (χ^2^) was used to compare clinical and neurological characteristics and frequencies of HRCs between SBO and SBA patients. Comparisons among continuous variables were done by a one-way ANOVA test. Separate comparisons between two different groups of continuous variables were done using a Mann–Whitney U test. The Pearson correlation test was used for the assessment of the correlation between the degree of neurogenic lesion and the degree of genetic homozygosity, where r values between 0.1 and 0.3 were classified as small, between 0.3 and 0.5 classified as medium and above 0.5 as large [23]. The variation coefficient (CV) was used to present the genetic homozygosity variability of tested groups of individuals. The statistical significance was set at *p* < 0.05.

## 3. Results

In Table 1, we presented the test and retest results of interrater reliability for tested HRCs, between two testing persons that have tested individuals from the control sample. On all three occasions, we achieved almost perfect agreement, whereas on the second retesting for 14 HRCs, the agreement was 1 (perfect), while for one HRC, the disagreement was for one observation out of 100, leading to kappa −0.98, and still representing an almost perfect agreement.

There were 76 patients with SBO and 46 with SBA. The predominant type of neurogenic lesion in the entire group of SB patients was paresis (49.2%), where it was found that every second participant had such lesion, while paralysis was less frequent (18%) (Table 2). Paralysis was noticed only in patients with SBA, while non-significant distribution in frequencies of paresis between SBO and SBA were found (*p* = 0.6069) (Table 2). 

**Figure 1:**
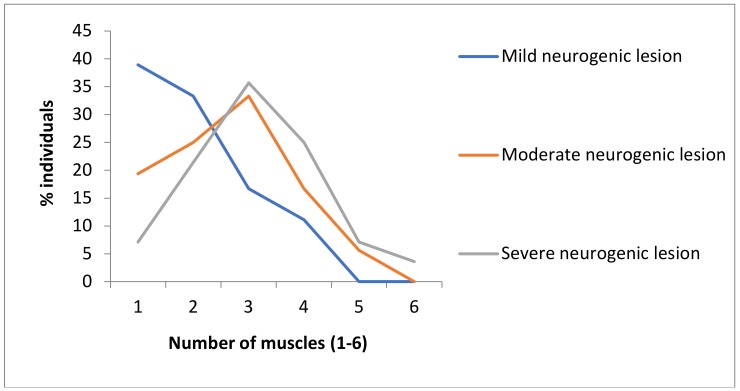
Frequencies of affected muscle groups in groups of patients with different degrees of neurogenic lesion (MV ± SD). Mild neurogenic lesion group (*N* = 18) $\bar{x}$ = 2.0 ± 1.0; moderate neurogenic lesion group (*N* = 36) $\bar{x}$ = 2.6 ± 1.2; severe neurogenic lesion group (*N* = 28) $\bar{x}$ = 3.1 ± 1.2.

There were 18 SB patients with mild degrees, 36 with moderate degrees and 28 with severe degrees of neurogenic lesion. The most frequent type of neurogenic lesion was moderate, close to half (43.9%) and least frequent mild degree, just above every fifth SB patient (22%) (Figure 1). It is pointed out that, as a neurogenic lesion is more severe, the number of affected muscles starts to increase, which is obvious, especially between groups with mild and moderate neurogenic lesions and between groups with mild and severe neurogenic lesions (Figure 1; Table 3).

**Figure 2:**
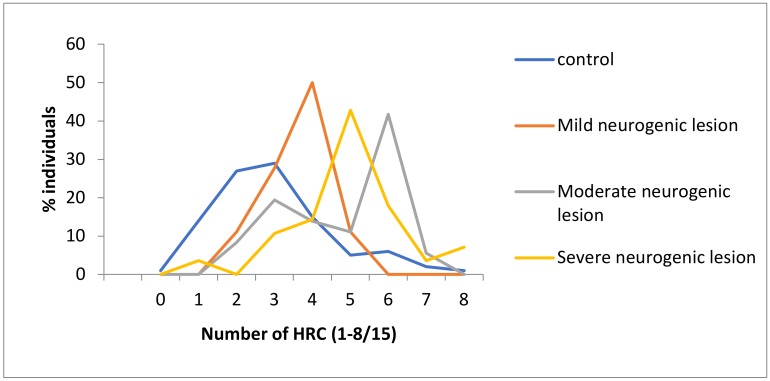
Frequencies of homozygous recessive characteristics (HRC) in different groups of patients with spina bifida and control (MV ± SEM). Control $\bar{x}$ = 3.0 ± 0.2; Total $\bar{x}$ = 4.6 ± 0.2; Mild degree $\bar{x}$ = 3.6 ± 0.2. Moderate degree $\bar{x}$ = 4.8 ± 0.3. Severe degree $\bar{x}$ = 5.0 ± 0.3.

Our results pointed out that as neurogenic lesions are more severe, HRC values start to increase (control group $\bar{x}$_HRC/15_ = 3.0 ± 0.2, CV = 53.3%; mild neurogenic lesion group $\bar{x}$_HRC/15_ = 3.6 ± 0.2, CV = 23.5%; moderate neurogenic lesion group $\bar{x}$_HRC/15_ = 4.8 ± 0.3, CV = 32.0%; severe neurogenic lesion group $\bar{x}$_HRC/15_ = 5.0 ± 0.3, CV = 29.8%), indicating an increase in individual genetic homozygosity, as well as a decrease in variability (CV_Control_ = 53.3%; CV_SB_ = 32.2%) (Figure 2). The Pearson correlation demonstrated that there is a positive medium to moderate correlation between the degree of neurogenic lesion and the degree of genetic homozygosity (r = 0.320), indicating that an increase in the degree of neurogenic lesions is followed by an increase in the degree of genetic homozygosity (Table 3).

## 4. Discussion

Neural tube closure is a process involving interactions between genes, where environmental factors play significant role, pointing out the multifactorial inheritance of the spina bifida entity [24]. Beside the environmental factors that were studied broadly in several studies [8,24], as well as some molecular genetic observations, including the correlation of certain mutations on X chromosome and its influence on the SB formation [3,10,25,26,27,28], a population-genetic approach, as a new method, can give a new insight on etiology for individuals with spina bifida [4,29].

Our findings demonstrated that the most frequent form of neurogenic lesion was one that was defined as moderate degree. Further, in the group of patients with SBO, paresis was presented in almost half of evaluated children, while there were no patients with paralysis. For the group of participants with diagnosed SBA, the most frequent form of clinical manifestations of neurogenic lesion was paresis, presented in more than half of the children. Investigations in hatched chick models have suggested that there might be a smaller number on interneurons in SBA chicks in segments innervating dysfunctional muscles, and thus resulting in leg dysfunctions [30]. This might bring to assumption that there could be some type of more sensitive predisposition for leg dysfunctions in SBA. The fact that there is a significantly higher percentage of patients with paralysis in the group of SBA affected individuals (compared to SBO) points out the more severe degree of expression of such an entity. 

In the group with a mild degree of neurogenic lesion, one third of the tested muscles were affected. The group with moderate neurogenic lesions showed a higher number of affected muscles (close to half), while in the group with severe neurogenic lesions, more than half of the evaluated muscles were affected. These results clearly indicate that as neurogenic lesion is more severe, more muscles that are innervated by nerve roots from the lumbosacral spine region are affected, and a basis of a greater recessive genetic homozygosity is present. 

According to the data presented in this study, the frequency distribution of the tested HRCs was clearly different in compared groups of individuals, manifesting in a population genetic difference that exists among them. It can be seen that the average percentage of recessive homozygosity in the control group of individuals is 20% ($\bar{x}$_hrc/15_ = 3.0 ± 0.2), in the group of individuals with mild neurogenic lesion this is around 25% ($\bar{x}$_hrc/15_ = 3.6 ± 0.2), and finally, in the groups of individuals with moderate and severe neurogenic lesions, it is more above than 30% (moderate neurogenic lesion group-$\bar{x}$_hrc/15_ = 4.8 ± 0.3; severe neurogenic lesion group-$\bar{x}$_hrc/15_ = 5.0 ± 0.3). These findings point out that an increase in the degree of neurogenic lesions is followed by an increase in genetic homozygosity, as well as a decrease of the genetic variability for tested characteristics in affected groups of individuals.

There are several possible explanations for the established differences in recessive homozygosity degree among different groups of affected individuals and the control [12,13,15,29,31]:

- An increase in individual genetic homozygosity, as well as a decrease in genetic variability in affected groups of individuals, may bring an organism into a specific state of genetic–hysiological homeostasis, which enables easier expression of different degrees of neurogenic lesions in SB patients.
- An increase in the genetic homozygosity degree may raise genetic loads, thus potentially causing decreased body resistance to developmental disbalances, respectively rising the expression of recessive genes that might be related to spina bifida.

A higher degree of genetic homozygosity may result in pleiotropic effects of specific genes responsible for the expression of illness. Those genes will not only determine the expression of SB, but also a group of other characteristics, including HRC properties, and this is illustrated in several of our previous studies.

Finally, we have demonstrated larger individual variations in tested HRCs for SB patients with severe neurogenic lesions (from 1-8), versus those with mild forms (from 2–5), pointing to the fact that large variations in genetic homeostasis, which might exist in human individuals, could raise the susceptibility of exposure of extreme genotypes to the risk of suffering from defined developmental and metabolic malformations [13,16].

There are several limitations to this study. The first limitation refers to the study sample from the single population, where specific pleiotropic variations in spina bifida expression might exist in different populations, and thus a multipopulational approach is advised for future investigations. Moreover, the number of studied individuals might be taken as a limiting factor. 

## 5. Conclusions

It is difficult to evaluate the boundaries of normal genotypic variation in humans, but it is possible to predict the circumstances to which a group of variants could be exposed in the near future, regarding some health problems. Our study demonstrated that an increased degree of recessive homozygosity for tested HRCs implies a higher likelihood of having spina bifida. Therefore, the applied methodology that we used in this study could give insight into the wider understanding of the multifactorial etiology of spina bifida, and thus be proposed as a potential screening method for spina bifida in humans.

## Figures and Tables

**Table 1:** Interrater reliability of homozygously recessive characteristics.

Homozygously Recessive Characteristics	Kappa Value (Control Sample *N* = 100)		
	Test	Retest - 1	Retest - 2
Blond Hair	0.94	0.96	0.98
Straight Hair	0.98	1.00	1.00
Double Hair Whorl	1.00	1.00	1.00
Opposite Hair Whorl Orientation	0.96	1.00	1.00
Soft Hair	0.96	0.98	1.00
Continuous Hair Line	0.96	1.00	1.00
Attached Ear Lobe	0.96	1.00	1.00
Ear Without Darwinian notch	1.00	1.00	1.00
Blue Eyes	0.96	1.00	1.00
Color Blindness	1.00	1.00	1.00
Right Thumb over Left Thumb	1.00	1.00	1.00
Hand Clasping Pattern	1.00	1.00	1.00
Proximal thumb extensibility	0.98	1.00	1.00
Left-handedness	1.00	1.00	1.00
Index finger longer than the ring finger	0.98	1.00	1.00

**Table 2:** Distribution of clinical and neurological characteristics of patients with spina bifida.

Spina Bifida	Total	Paralysis		Paresis	
	*N*	*N* (%)	p*	*N* (%)	p*
SBO	76	0 (0)	<0.0001	36 (47.4)	0.6069
SBA	46	22 (47.8)		24 (52.2)	
Total	122	22 (18)	-	60 (49.2)	-

SBO = spina bifida occulta; SBA = spina bifida aperta; *—χ^2^ test.

**Table 3:** Statistical analysis of HRC and affected muscles’ proportion regarding degree of neurogenic lesions.

	HRC		Affected Muscles	
	*p* *	*p* **	*p* *	*p* **
All groups	-	<0.0001	-	<0.0054
Mild and Moderate	0.0093	-	0.0615	-
Moderate and Severe	0.9045	-	0.1211	-
Mild and Severe	<0.0005	-	0.0029	-
Mild and control	0.0160	-	-	-
Moderate and control	<0.0001	-	-	-
Severe and control	<0.0001	-	-	-
Degree of neurogenic lesion/Degree of genetic homozygosity	Pearson correlation			
	0.320			

HRC= homozygous recessive characteristics; *—Mann–Whitney U test; **—One-way ANOVA test.
